# Lung cancer patients’ comorbidities and attendance of German ambulatory physicians in a 5-year cross-sectional study

**DOI:** 10.1038/s41533-020-00214-8

**Published:** 2021-01-28

**Authors:** Jasmin Bossert, Marion Ludwig, Pamela Wronski, Jan Koetsenruijter, Katja Krug, Matthias Villalobos, Josephine Jacob, Jochen Walker, Michael Thomas, Michel Wensing

**Affiliations:** 1 Department of General Practice and Health Service Research, University Hospital Heidelberg, Im Neuenheimer Feld 130.3, 69120 Heidelberg, Germany; 2 InGef - Institute for Applied Health Research Berlin GmbH, Spittelmarkt 12, 10117 Berlin, Germany; 3 Department of Thoracic Oncology, University Hospital Heidelberg and Translational Lung Research Centre Heidelberg (TLRC-H), Member of the German Centre for Lung Research (DZL), Heidelberg, Germany

**Keywords:** Health services, Respiratory tract diseases

## Abstract

The majority of lung cancer patients are diagnosed with an advanced stage IV, which has short survival time. Many lung cancer patients have comorbidities, which influence treatment and patients’ quality of life. The aim of the study is to describe comorbidities in incident lung cancer patients and explore their attendance of ambulatory care physicians in Germany. In the observed period, 13,111 persons were first diagnosed with lung cancer (1-year incidence of 36.4 per 100,000). The mean number of comorbidities over 4 quarters was 30.77 ± 13.18; mean Charlson Comorbidity Index was 6.66 ± 2.24. In Germany, ambulatory care physicians most attended were general practitioners (2.6 quarters with contact within 4 quarters). Lung cancer was diagnosed by a general practitioner in 38% of the 13,111 incident patients. The average number of ambulatory care physician contacts over 4 quarters was 35.82 ± 27.31. High numbers of comorbidities and contacts in ambulatory care are common in patients with lung cancer. Therefore, a cross-sectoral and interdisciplinary approach is required for effective, patient-centred care. This was a 5-year cross-sectoral study, based on the InGef research database, which covers anonymized health insurance data of 7.2 million individuals in Germany. Incident lung cancer patients in a 5-year period (2013–2017) were identified. Descriptive statistics were calculated for sociodemographic characteristics, comorbidities, and attendance of ambulatory care physicians.

## Introduction

Despite improved diagnostic and treatment options, lung cancer remains one of the most malignant diseases and the leading cause of cancer-related deaths worldwide^1^. The majority of lung cancer patients (about 70%) are diagnosed with an advanced stage IV disease, which is characterized by short survival time in comparison with other cancer diseases (the 5-year survival in Europe is <20%)^2^. Comorbidities seem to influence cancer prognosis survival in various ways^3^. The prevalence of comorbidity increases with age^4^. Due to the fact that lung cancer is most frequent in men in the age of 80–84 years and women in the age of 70–74 years, consideration of comorbidities during treatment planning should receive more attention^5^. Most clinical guidelines of cancer treatment do not reflect the complex inter-relation between cancer and comorbidity but instead pursue a “single disease” approach in management^6^. Nevertheless, diseases that existed before cancer diagnosis have an effect on the risks and outcomes of cancer treatments, both in cancer in general and specifically in lung cancer^7,8^. Therefore, data on comorbidities, along with patient demographics, is essential for comprehensive treatment and care in lung cancer. Such data are required to enhance clinical decision-making and supportive tools, as well as the design and planning of healthcare services for patients with lung cancer^6,8,9^.

With the growing complexity in the treatment of cancer and comorbidities, cross-sectoral and interdisciplinary coordinated patient care has become the preferred model in health care^10^. In many situations, it is necessary to set priorities in the treatment and care of different diseases of a patient, which requires effective cooperation between providers in different healthcare settings^11^. Furthermore, it offers patients the possibility to get a balanced perspective on the risks and benefits of all available treatment options during treatment decision, thus strengthening prognostic awareness. To reach effective cross-sectoral cooperation, current data on the prevalence of comorbidities and the contacts with various healthcare providers involved is required^12^.

To our knowledge, there are no studies in Germany that document comorbidities in lung cancer patients and the attendance of ambulatory care physicians involved in the treatment for lung cancer patients. The aim of the present study was to describe the comorbidities in newly diagnosed lung cancer patients in Germany and explore the physicians they consulted in ambulatory care.

## Results

### Prevalence

Out of the sample of 4.9 million patients, 3,275,709 were constantly insured (with the insurers linked to the database), and of these, 20,802 persons (mean age 70 years) had the diagnosis lung cancer in the period 2013–2017. This corresponds to a 5-year prevalence rate of 635 persons per 100,000 inhabitants with 1-year prevalence rates between 206.6 (year 2013) and 227.8 (year 2016) per 100,000 persons. Supplementary Table 1 presents an overview of prevalent cases from 2013 to 2017.

### Study population (incident cases)

In the period 2013–2017, 13,111 persons (mean age 70 years) were newly diagnosed with lung cancer. Numbers varied per year between 2649 and 2826 patients corresponding to incidence rates of 69.1 (95% confidence interval (CI) 66.45–71.73) and 76.6 (95% CI 73.84–79.52) per 100,000 persons (Table 1). More men (*n* = 8301; 63.31%) than women (*n* = 4810; 36.69%) were affected (Table 2). For the purposes of healthcare services research, a sample of approximately 4 million patients per year was selected, which is representative of the German population regarding age and sex. Projected to the total German population, an estimated 57,070–62,135 persons per year was diagnosed with lung cancer between 2013 and 2017.

**Table 1:** Incidence of lung cancer (C34*).

	Fully insured persons	*N*	*N* non-prevalent	Cases/100,000	Lower 95% CI bound	Upper 95% CI bound
	*N*					
2013	3,649,007	2660	3,643,814.00	73.00	70.25	75.83
2014	3,692,998	2826	3,687,290.00	76.64	73.84	79.52
2015	3,780,098	2810	3,774,074.00	74.46	71.73	77.26
2016	3,799,249	2832	3,793,060.00	74.66	71.94	77.46
2017	3,842,919	2649	3,836,387.00	69.05	66.45	71.73
2012–2017	3,129,423	13,111	3,122,023.00	419.95	412.81	427.19

**Table 2:** Incidence of lung cancer stratified by age and sex.

	2013		2014		2015		2016		2017		2013–2017	
	*N*	%	*N*	%	*N*	%	*N*	%	*N*	%	*N*	%
Incident patients												
Number of patients	2660	100	2826	100	2810	100	2832	100	2649	100	13,111	100
<60 years	887	33.4	891	31.5	887	31.6	886	31.3	775	29.3	3095	23.6
60–75 years	1599	60.1	1751	62.0	1718	61.1	1746	61.7	1666	62.9	8528	65
>75 years	174	6.5	184	6.5	205	7.3	200	7.1	208	7.9	1488	11.4
Male												
Number of patients	1710	100	1774	100	1817	100	1723	100	1646	100	8301	100
<60 years	499	29.2	490	27.6	523	28.8	474	27.5	438	26.6	1713	20.6
60–75 years	1099	64.3	1176	66.3	1165	64.1	1,125	65.3	1076	65.4	5629	67.8
>75 years	112	6.6	108	6.1	129	7.1	124	7.2	132	8	959	11.6
Female												
Number of patients	950	100	1052	100	993	100	1109	100	1003	100	4810	100
<60 years	388	40.8	401	38.1	364	36.7	412	37.2	337	33.6	1382	28.7
60–75 years	500	52.6	575	54.7	553	55.7	621	56	590	58.8	2899	60.3
>75 years	62	6.5	76	7.2	76	7.7	76	6.9	76	7.6	529	11

### Comorbidities

Essential (primary) hypertension was the most frequent comorbidity of incident lung cancer patients in Germany (74.3%) without substantial differences between the different federal states (range: 62.8–84.4%) (Supplementary Table 2). The second most frequent comorbidity in Germany was “disorders of lipoprotein metabolism and other lipidomes” (51.4%) followed by “other chronic obstructive pulmonary diseases” (46.6%). In total, 1326 different comorbidities were detected at the 3-digit level during the observation period (patient-individual year). A comprehensive list of the 100 most frequent comorbidities can be found in Supplementary Table 3. The average number of comorbidities (including all co-occurring diseases) within four quarters is higher in women than in men and is highest in the age group >75 years (women: 33.93 ± 13.88; men: 32.65 ± 13.35). In both sexes and in all age groups, the Charlson Comorbidity Index (CCI) is about 6 (range: 6.09–6.97) (Table 3).

**Table 3:** Average number of comorbidities and mean CCI score of incident lung cancer patients.

	Average number of comorbidities		Charlson Comorbidity Index	
	Mean	SD	Mean	SD
Incident patients				
All	30.77	13.17	6.66	2.24
<60 years	27.10	12.39	6.20	2.04
60–75 years	30.49	12.89	6.62	2.20
>75 years	33.06	13.54	6.96	2.35
Male				
<60 years	26.42	12.45	6.29	2.06
60–75 years	30.14	12.73	6.70	2.24
>75 years	32.65	13.35	6.97	2.36
All	30.49	13.07	6.73	2.27
Female				
<60 years	27.94	12.27	6.09	2.01
60–75 years	31.07	13.14	6.49	2.13
>75 years	33.93	13.88	6.95	2,35
All	31.26	13.34	6.54	2.19

### Ambulatory care physicians

Both in Germany overall (mean = 2.6 contact quarters (CQs) within 4 patient-individual quarters) as well as in the individual German federal states (mean = 2.4–2.8), the general practitioner was contacted most frequently, followed by internists with an average of 2.3 contact in Germany and an average ranging from 2.0 to 2.4 in the individual federal states. The ear, nose and throat specialists (ENT) were contacted on average in 1.4 quarters (see also Supplementary Table 4), which is linked to the most common comorbidities of incident lung cancer patients (Supplementary Table 2), as chronic obstructive pulmonary diseases and respiratory insufficiency are also treated by the ENT. Within the observation period of four patient-individual quarters, the average number of visits to ambulatory care physicians in incident lung cancer patients was higher for women aged <60 years (45.08 ± 30.26) and women aged between 60 and 75 years (38.51 ± 27.30) than for men in this age groups (40.38 ± 29.24/36.10 ± 27.06). In the age group >75 years, it can be seen that men (31.57 ± 25.93) had a slightly higher number of contacts with ambulatory care physicians than women (28.60 ± 23.47) (Table 4).

**Table 4:** Average number visits to ambulatory care physicians.

	Average number of visits	
	Mean	SD
Incident patients		
All	35.82	27.31
<60 years	42.49	29.79
60–75 years	36.99	27.17
>75 years	30.62	25.21
Male		
<60 years	40.38	29.24
60–75 years	36.10	27.06
>75 years	31.57	25.93
All	35.13	27.15
Female		
<60 years	45.08	30.26
60–75 years	38.51	27.30
>75 years	28.60	23.47
All	37.01	27.54

Table 5 shows the specialists who first diagnosed lung cancer in the outpatient sector. The analysis shows that the general practitioner most often made the initial diagnosis in the outpatient sector (38.1%). In 6.7% of all cases, the specialty of the diagnosing physician was not registered. The majority of patients seem not to be diagnosed in the outpatient sector but by inpatient healthcare providers.

**Table 5:** Specialists who first diagnosed lung cancer in the outpatient sector.

Specialist group	Patients (*n*)	(%)
General practitioner	4995	38.1
No information	874	6.7
Pulmonologist	335	2.6
Haematologist/Oncologist	269	2.1
Ophthalmologist	224	1.7
Urologist	151	1.2
Orthopaedic	150	1.1
ENT specialist	132	1.0
Nuclear medicine specialists	130	0.9
Radiologist	122	0.9

## Discussion

This study documented the number of incident lung cancer patients in Germany and their most frequent and number of comorbidities, the Charlson Comorbidity Index, contacts to ambulatory care physicians, differences between federal states and ambulatory care physicians who made the initial diagnosis of lung cancer in Germany in incident lung cancer patients. Lung cancer patients have many comorbidities (30.77 ± 13.18) and a high CCI score, indicating major burden from multimorbidities. Many of the most prevalent comorbidities are in the domain of internal medicine. The specialist group most consulted by lung cancer patients in ambulatory care is the general practitioner. General practitioners also make the initial diagnosis most frequently throughout Germany.

Data from the Robert-Koch-Institute (based on the population-based cancer registry in Germany) also show that men (36,000) are more frequently diagnosed with lung cancer than women (21,500). This corresponds to a 1-year incidence rate for men of 88.6/100,000 and for women of 51.5/100,000, which is comparable to our results. The prevalence rates found in this study are higher than the official cancer registry data in Germany^5^. This could be explained by the fact that only a limited number of statutory health insurance (SHI) organizations were included in this analysis.

About 75% of the patients included in our study had at least one comorbidity. The average number of comorbidities in incident lung cancer patients was 30.77, measured over four patient-individual quarters. However, it is important to note that each co-existing disease is included in the analysis (including single-event diagnoses, such as cystitis), which leads to the relatively high number of average comorbidities over four quarters. The high number of comorbidities can also be explained by the fact that the underlying data structure of this study is very differentiated and uses the 3-digit tenth revision of the International Classification of Diseases (ICD)-10 code with its highly specific breakdown of diseases of one organ. For example, a given intestinal disease may have received two different codes within a patient-specific year, which could have been aggregated in other studies with a significantly lower number of comorbidities^13,14^. Despite this fact, it is reasonable to assume that the average number gives an indication that lung cancer patients suffer from a disease burden that requires expertise from various medical domains, even if not all included co-existing diseases progress to a permanent diagnosis. On the other hand, the aspect of death as an exit from the study sample has to be considered. Even if the mortality of the included patients has not been analysed, it is still important to keep this in mind with regard to the average number of comorbidities. This might have reduced the average number of comorbidities.

The most common comorbidity is essential primary hypertension. Studies show that the prevalence of essential primary hypertension is nationwide on a high level, regardless of whether an oncological diagnosis is available. The prevalence of hypertension increases with age. Almost two-thirds of Germans aged ≥65 years have a diagnosed hypertension^15^.

The number of CQs and the average number of visits to ambulatory care physicians gives an estimate of the extent of comorbidities requiring treatment. Studies show that the severity of an illness affects the number of contacts with care physicians^16^. Evidence from 2020 demonstrate that comorbidities in lung cancer patients lead to >50% annual rise in the number of contacts to ambulatory care physician. Furthermore, a German study reflects that every additional comorbidity to an index disease is associated with an increase by the factor of 2.3 in medical contacts. Consequently, the number of comorbidities is associated with a significant increase in physician contacts compared to patients without comorbidity^17^. Considering that in 2007 the number of physician contacts for all statutory insured people in Germany was 17^18^, the disease burden of patients with lung cancer is underlined by the fact that they visited an outpatient care provider 35 times and have a CCI about 6.

However, general practitioners and most other specialist groups are not comprehensively skilled to manage the wide spectrum of coexisting diseases in patients with cancer. Lung cancer patients with comorbidities often need multimodal treatment and care, delivered by different professionals, in different settings and at different time points. In order to obtain complete information on comorbidities, all involved specialist groups providing treatment for lung cancer patients with comorbidities should be mutually known^6^. At the same time, clinicians outside oncology (including ambulatory care physicians) need to consider the impact of cancer in their treatment decisions on comorbid diseases. This requires effective cross-sectoral cooperation^12^. To achieve the goal of cross-sectoral and multidisciplinary coordinated patient care, an effective network of all specialists involved within one sector is a precondition.

Within the network of cross-sectoral cooperation, current evidence indicates that a coordinator should be appointed to organize and priorities healthcare processes across service sectors^19,20^. In this context, the general practitioner is usually the first person to be contacted by patients with health problems and is therefore conceivable in the role of a coordinator^21^. In addition, the general practitioner is the care provider who generally has a long-term relationship with patients, which is often intensified following a cancer diagnosis^22^.

Furthermore, patients with advanced cancer often experience prolonged and frequent hospital stays^22^. Between hospital stays, patients are treated by outpatient specialists for both cancer and comorbidities. Cooperation and effective networking of all stakeholders, both within a sector and across sectors, supports patient-centred care.

A conceivable approach to support cross-sectoral cooperation could be the use of an electronic platform. The central access to patient-relevant data facilitates the exchange between participating specialists and also has a positive impact on organizational aspects, since the personal exchange of information is not fundamental and can be reduced to special events in the treatment context. In particular, general practitioners in their role of the treatment coordinator could benefit from the use of electronic tools such as the electronic patient record as they can quickly and easily provide anamnestic information for other treating specialists and receive information on activities from other physicians. In this way the cooperation between the participating service providers might be improved^23^. Considering the average number of comorbidities, the CCI scores (>5), which are associated with a high risk of death within the next year, and the contacts to care providers, this aspect is gaining in relevance and can help to improve patient safety. Treatment errors can be avoided by providing comprehensive digital patient data for healthcare providers, especially in emergency situations. Furthermore, there is a positive correlation between the electronic recording of medication and patient adherence^24^.

Although the use of electronic patients’ records could be a solution to overcome barriers in cross-sectoral healthcare, their implementation is still a major challenge^25^. One of the reasons for the difficulties in implementing telemedicine is the very restrictive data protection laws in Germany. These guarantee a high level of data security for patients but currently offer limited scope for innovation^26^. An additional challenge in implementing e-health across healthcare sectors is the interoperability of the currently existing data systems. Accordingly, serious problems often arise when integrating different electronic systems within the outpatient and inpatient sector. To solve the problem, the uncontrolled growth of different standard operating procedures in the file systems should be eliminated. In addition to interoperability as a central prerequisite for digitization, government policy is seen as a relevant component. Therefore, it is the task of politics to improve the necessary framework conditions for the successful implementation of e-health in the healthcare system to support healthcare across service sectors^25^. Furthermore, the users’ attitude towards e-health must be considered. Therefore, the usability of new technologies is perceived as an additional challenge by some individuals^27^.

In summary, treating patients with oncological diagnoses involves consideration of their comorbidities, the involvement of other treating specialists and their treatment approaches. It must be aimed to develop a common therapy plan that reflects all the existing diseases of a patient and is coordinated with all treating physicians. The results obtained in this study serve as an indicator for the treatment of lung cancer patients and justify the need for collaborative treatment planning. The results of the analysis show that lung cancer patients consult not only general practitioners but also various other specialists (e.g. outpatient internists). Effective cross-sectoral cooperation is seen as an improvement in patient safety and health-related quality of life. Especially for diseases with a limited prognosis, which are associated with a high risk of mortality within the next year, the relevance of these aspects increases.

Comorbidities are frequent in patients with lung cancer, which leads to the assumption that there is much potential for improvement in coordination for the individual patient. Therefore, a complete assessment of comorbidities and ambulatory care physicians is needed for a holistic treatment approach and contributes to patient-centred care. A cross-sectoral and interdisciplinary approach based on a uniform electronic platform is recommended.

Limitations of this analysis include those inherent to claims database studies, such as the reliance on accurate coding and diagnosis. Consequently, results need to be interpreted cautiously. In general, routine data are collected primarily for reimbursement purposes and not for clinical research. For example, the data of privately insured persons may differ from that of persons with SHI organizations due to different reimbursement practices or with regard to their socioeconomic status and therefore the generalizability of the results obtained in this study may be limited^28^. Nevertheless, the validity and comprehensiveness of German health claims data can be considered very high. The fact that only a limited number of SHI organizations were included in this analysis could explain why the calculated prevalence rate of this study shows large deviations to the prevalences according to the official cancer registry data in Germany^5^.

## Methods

### Study design

This study is a 5-year cross-sectoral study using claims-based data. The observation period for identifying incident lung cancer patients was 01.01.2012–31.12.2017. Incident cases of lung cancer patients were determined for a 5-year period (01.01.2013–31.12.2017 inclusion period; 01.01.2012–31.12.2012 additional lookback time) to report age, gender, the ten most frequent comorbidities, the ten most involved ambulatory care physicians, number of quarter years with contact to ambulatory care physicians’ groups and differences between federal states in Germany.

### Data source

We used data from the InGef (Institute for Applied Health Research Berlin GmbH) research database. This database is an anonymized healthcare claims database with longitudinal data from approximately 7.2 million persons (8.67% of the German population), who are insured with one of >60 German SHI organizations. Data since 2012 are available in this database. For this study, a sample of 4.9 million patients from January 2013 to December 2017 was drawn and adjusted for age and sex based on official national statistics (Destatis, 31 December 2017).

All diagnoses in the database are coded according to the German modification of the ICD-10 (ICD-10-GM). All patient-level data in the InGef database are de-identified to comply with German data protection regulations and German federal law. Hence, separate approval of an institutional review board or Medical Ethics Committee was not required. In brief, the database includes the following data: an anonymized identification number, demographic data (age, sex, region of living), date of death, periods of coverage for the health insurance, diagnoses according to ICD-10-GM for ambulatory care and hospital data, including admission and discharge date, procedures and diagnoses, prescription data and specialty of the prescribing or diagnosing physician^29^.

### Study population

Adult patients (≥18 years) were only included in the study population (incident cases) if they were continuously insured during the observation period at one of the SHI organization included in the InGef database. Patients were included if they met the following inclusion criteria: A newly assured diagnosis of ICD-10-GM C34* in the outpatient sector or main and secondary diagnosis of ICD-10-GM C34* at discharge in the inpatient area. A diagnosis was considered confirmed if the diagnosis ICD-10-GM C34* in the outpatient sector was mentioned by two different physicians in the index quarter (quarter of the initial diagnosis) or by the same physician in two consecutive quarters. The unique diagnosis of ICD-10-GM C34* made by providers of the inpatient sector was also regarded as a confirmed diagnosis.

An incident lung cancer case entered the study sample at the moment of the first diagnosis following a validation period of 4 quarters (2012) free of lung cancer diagnoses to ensure that only incidences of lung cancer were present. Re-entry into the study sample by the same patient was not allowed. Study entry was at the beginning of the quarter in which lung cancer diagnosis was first documented. Study exit was defined as death or the end of the inclusion period (31.12.2017). There could also be incidence patients in the study sample who died within the follow-up period. Of note, these patients then contribute correspondingly less to the average comorbidity or quarterly number of specialist visits.

### Measures

The measures concerned comorbidities and involved ambulatory care physicians in incident lung cancer patients.

Comorbidities were defined as confirmed outpatient diagnoses (3-digit) and/or inpatient primary and/or secondary diagnoses (3-digit) according to ICD-10-GM. The classifications O (pregnancy, birth, postpartum) and Z (facts influencing the state of health and leading to the use of health services) were excluded as they do not indicate diseases. Comorbidities were identified during four quarters: the quarter before the first documentation of the lung cancer diagnosis, the quarter in which the lung cancer diagnosis was made and two quarters after the initial lung cancer diagnosis (see Supplementary Table 5 for a thorough description of all measures included). These four quarters are described in this paper as patient-individual years. Comorbidities were only considered once within the patient-individual year even if they were documented more than once. The CCI was also calculated for the four patient-individual quarters and includes 19 different diseases^30^. Each comorbid disease ranges from 1 to 6 points to summate an index value. The index value enables the division into four prognostically significant categories (0, 1–2, 3–4, ≥5)^31^. Accordingly, the annual mortality of category 0 is 12 and 85% for category 5.

Individual ambulatory care physicians and their specialist area were identified by the Lifelong Physician Identification Number. All outpatient diagnoses contain the information in which physician documented the outpatient diagnosis. For patients whose diagnosis of a concomitant disease was documented by two different ambulatory care physicians in the index quarter, both specialists were registered, since it is not possible to distinguish who made the initial diagnosis. We included all relevant specialists for the treatment of oncological diseases in the field of internal medicine.

The German Medical Association defines the conditions under which specialists in internal medicine (internists) and general medicine (general practitioners) can acquire the specialist or specialization title^32^.

Additionally, patient’s age and the number of quarters with at least one contact to a specific ambulatory care physician group were registered. Age was calculated as the age on 31 of December in the previous year and as the age on 31 December 2017 for the 5-year prevalence. Next, patients were assigned to three age groups: <65, 65–85, and >85 years (Additional File 1). To calculate the number of quarters with at least one contact to a specific ambulatory care physician group, per patient, four individual quarters were observed (patient-individual year). This means that the maximum number of CQs to outpatient physicians is four. The same time frame was used to calculate the average number of visits to ambulatory care physicians.

### Data analysis

For the years 2013 to 2017, 5-year prevalence and 1-year prevalences of lung cancer were calculated. Incidence for 2013 to 2017 was calculated using 2012 as validation period. Prevalence and incidence crude rates were adjusted by age and gender distribution of the German population in the respective year or on 31.12.2017 for 5-year estimates. According to clinical experience, the diagnosis of lung cancer generally becomes a permanent diagnosis and no full recovery is possible. Permanent diagnoses are available on a quarterly basis.

To describe the sociodemographic and clinical characteristics of incident lung cancer patients, mean (M), standard deviation (SD), median (Md), minimum (min) and maximum (max) were calculated for continuous variables (definitions and description of all variables in Additional File 1). For binary variables, absolute (*n*) and relative (%) frequencies of patients were specified. Regional differences are presented stratified annually from 2013 to 2017 in order to observe changes over a period of 5 years. In this context, a descriptive and non-standardized analysis of age and gender distribution was performed. For descriptive and comparative purposes, 95% CIs are provided assuming a Poisson distribution. All statistical analyses were conducted using Microsoft R Open 3.5.0.

### Reporting summary

Further information on experimental design is available in the Nature Research Reporting Summary linked to this paper.

## Supplementary information

Supplementary information

Reporting summary

## Data Availability

The data used in this study cannot be made available in the manuscript, the supplemental files or in a public repository due to German data protection laws (Bundesdatenschutzgesetz). To facilitate the replication of results, anonymized data used for this study are stored on a secure drive at the Institute for Applied Health Research Berlin (InGef). Access to the data used in this study can only be provided to external parties under the conditions of the cooperation contract of this research project and can be assessed upon request, after written approval (info@ingef.de), if required.

## Code availability

Available upon request.

## References

[1] Grønberg BH (2010). Influence of comorbidity on survival, toxicity and health-related quality of life in patients with advanced non-small-cell lung cancer receiving platinum-doublet chemotherapy. Eur. J. Cancer.

[2] Eberle A (2015). Lung cancer survival in Germany: a population-based analysis of 132,612 lung cancer patients. Lung Cancer.

[3] Grose D (2015). The impact of comorbidity upon determinants of outcome in patients with lung cancer. Lung Cancer.

[4] Gajra A (2016). Assessment of comorbidity in lung cancer: how, why, and in whom?. J. Geriatr. Oncol..

[5] Barnes, B. et al. Bericht zum Krebsgeschehen in Deutschland 2016 (Robert-Koch-Institut, 2016).

[6] Sarfati D, Koczwara B, Jackson C (2016). The impact of comorbidity on cancer and its treatment. CA Cancer J. Clin..

[7] Grose D (2014). Comorbidities in lung cancer: prevalence, severity and links with socioeconomic status and treatment. Postgrad. Med. J..

[8] Dutkowska AE, Antczak A (2016). Comorbidities in lung cancer. Pneumonol. Alergol. Pol..

[9] Islam KM, Jiang X, Anggondowati T, Lin G, Ganti AK (2015). Comorbidity and survival in lung cancer patients. Cancer Epidemiol. Biomarkers Prev..

[10] Engler J (2017). Physician cooperation in outpatient cancer care. An amplified secondary analysis of qualitative interview data. Eur. J. Cancer Care.

[11] Onukwugha, E. et al., editors. Specialist visits and initiation of cancer-directed treatment among a large cohort of men diagnosed with prostate cancer. *Urologic Oncology: Seminars and Original Investigations* **35**, 150.e17–150.e23 (Elsevier, 2017). 10.1016/j.urolonc.2016.11.012 28041997

[12] Baudendistel I (2017). Cross‐sectoral cancer care: views from patients and health care professionals regarding a personal electronic health record. Eur. J. Cancer Care.

[13] Eisele M (2018). Importance of comorbidities in the treatment of primary care patients with heart failure—baseline results of the observational RECODE-HF Study. Fam. Pract..

[14] Grønhøj C, Jakobsen KK, Kjær E, Friborg J, von Buchwald C (2019). Comorbidity in HPV+ and HPV− oropharyngeal cancer patients: a population-based, case-control study. Oral. Oncol..

[15] Neuhauser, H., Kuhnert, R. & Born, S. 12-Monats-prävalenz von bluthochdruck in Deutschland. *J. Health Monit*. **2**, 66–71 (2017).

[16] Kopetsch T, Maier W (2018). Analysis of the association between regional deprivation and utilization: an assessment of need for physicians in Germany. Gesundheitswesen.

[17] Ding R (2020). Comorbidity in lung cancer patients and its association with medical service cost and treatment choice in China. BMC Cancer.

[18] Kurth B-M, Lange C, Kamtsiuris P, Hölling H (2009). Gesundheitsmonitoring am Robert Koch-Institut. Bundesgesundheitsblatt-Gesundheitsforschung-Gesundheitsschutz.

[19] Green, E., Knight, S., Gott, M., Barclay, S. & White, P. Patients’ and carers’ perspectives of palliative care in general practice: a systematic review with narrative synthesis. *Palliat. Med*. **32**, 838–850 (2018). 10.1177/0269216317748862 29343169

[20] Weaver SJ, Jacobsen PB (2018). Cancer care coordination: opportunities for healthcare delivery research. Transl. Behav. Med..

[21] Lang V (2017). The role of the general practitioner in cancer care: a survey of the patients’ perspective. J. Cancer Res. Clin. Oncol..

[22] Kone, I., Klein, G., Siebenhofer, A., Dahlhaus, A. & Guthlin, C. GPs’ assessment of cooperation with other health care providers involved in cancer care-a cross-sectional study. *Eur. J. Cancer Care*10.1111/ecc.12751 (2018). 10.1111/ecc.12751 28983996

[23] Bauer, J., Rohner-Rojas, S. & Holderried, M. Consent management and workflows for cross-sectoral patient records and teleconsultations. *Radiologe* **60**, 430–439 (2020). 10.1007/s00117-020-00655-9 32060562

[24] Kaiser, M. J. & Fränken, J. in *Digitale Transformation von Dienstleistungen im Gesundheitswesen VI* 117–137 (Springer, 2019).

[25] Pohlmann S (2020). Digitalizing health services by implementing a personal electronic health record in Germany: qualitative analysis of fundamental prerequisites from the perspective of selected experts. J. Med. Internet Res..

[26] Koch R (2018). Improving cooperation between general practitioners and dermatologists via telemedicine: study protocol of the cluster-randomized controlled TeleDerm study. Trials.

[27] Waschkau, A., Flägel, K., Goetz, K. & Steinhäuser, J. Evaluation of attitudes towards telemedicine as a basis for successful implementation: a cross-sectional survey among postgraduate trainees in family medicine in Germany. *Evid. Fortbild. Qual. Gesundheits*. **156–157**, 75–81 (2020). 10.1016/j.zefq.2020.07.001 32859557

[28] Schmedt, N. et al. Pneumococcal vaccination rates in immunocompromised patients—a cohort study based on claims data from more than 200,000 patients in Germany. *PLoS ONE* **14**, e0220848 (2019). 10.1371/journal.pone.0220848 PMC6687114 31393931

[29] Andersohn F, Walker J (2016). Characteristics and external validity of the German health risk institute (HRI) database. Pharmacoepidemiol. Drug Saf..

[30] Charlson, M. E., Pompei, P., Ales, K. L. & MacKenzie, C. R. A new method of classifying prognostic comorbidity in longitudinal studies: development and validation. *J. Clin. Epidemiol*. **40**, 373–383 (1987). 10.1016/0021-9681(87)90171-8 3558716

[31] Wedding, U. & Schäffer, T. in *Geriatrische Onkologie* 27–36 (Springer, 2018).

[32] Bundesärztekammer. Arbeitsgemeinschaft der deutschen Ärztekammern. https://www.bundesaerztekammer.de/fileadmin/user_upload/downloads/20130628-MWBO_V6.pdf (2003).

