# Supplementary material for: Lung cancer patients’ comorbidities and attendance of German ambulatory physicians in a 5-year cross-sectional study
Source: NPJ Prim Care Respir Med. 2021 Jan 28;31:2. doi: 10.1038/s41533-020-00214-8 (PMC7844218; doi:10.1038/s41533-020-00214-8)
Supplement: Supplementary file 1 — Supplementary information [file 41533_2020_214_MOESM1_ESM.pdf]

Supplementary table 1: Prevalence of lung cancer stratified by age and sex

		2013		2014		2015		2016		2017		2013-2017	
		N	%	N	%	N	%	N	%	N	%	N	%
Prevalent patients	Number patients	7,979	100	8,657	100	8,987	100	9,167	100	9,347	100	20,802	100
	<60 years	2,558	32.1	2,730	31.5	2,754	30.6	2,737	29.9	2,696	28.8	4,668	22.4
	60-75 years	4,979	62.4	5,407	62.5	5,668	63.1	5,866	64.0	6,036	64.6	13,714	65.9
	>75 years	442	5.5	520	6.0	565	6.3	564	6.2	615	6.6	2,420	11.6
Male	Number patients	5,111	100	5,472	100	5,613	100	5,589	100	5,642	100	13,178	100
	<60 years	1,436	28.1	1,513	27.65	1,518	27.04	1,445	25.9	1,430	25.4	2,566	19.5
	60-75 years	3,392	66.4	3,631	66.4	3,745	66.7	3,793	67.9	3,817	67.7	9,039	68.6
	>75 years	283	5.5	328	6.0	350	6.2	351	6.3	395	7.0	1,573	11.9
Female	Number patients	2,868	100	3,185	100	3,374	100	3,578	100	3,705	100	7,624	100
	<60 years	1,122	39.1	1,217	38.2	1,236	36.6	1,292	36.1	1,266	34.2	2,102	27.6
	60-75 years	1,587	55.3	1,776	55.8	1,923	56,9	2,073	57.9	2,219	59.9	4,.675	61.3
	>75 years	159	5.5	192	6.03	215	6.4	213	5.9	220	5.9	847	11.1

Supplementary table 2: 10 most frequent comorbidities in incident lung cancer patients by federal state

	Essential (primary) hypertension	Disorders of lipoprotein metabolism and other lipidomes	Other chronic obstructive pulmonary disease	Secondary and unspecified malignant neoplasm of the lymph nodes	Secondary malignant neoplasm of the respiratory and digestive organs	Secondary malignant neoplasm at other and unspecified sites	Respiratory insufficiency not classified elsewhere	Other disturbances of the water and electrolyte balance and the acid-base balance	Back pain	Psychological and behavioural disorders caused by tobacco
Germany (n = 13,111)	74.3%	51.4%	46.5%	44.0%	39.9%	40.3%	37.6%	39.6%	38.4%	33.2%
North Rhine-Westphalia (n = 5,494)	75.6%	53.9%	47.9%	44.1%	39.8%	39.3%	39.3%	39.0%	38%	32.2%
Bavaria (n = 1,115)	70.5%	53.4%	46.5%	45.8%	41.6%	41.4%	38.7%	41.1%	42%	34.6%
Lower Saxony (n = 1,509)	72.8%	49%	48.2%	45.3%	38.2%	41.4%	31.1%	39.8%	39.3%	33.5%
Baden-Württemberg (n = 1,174)	70.7%	47.4%	39.3%	46.5%	43.9%	43.6%	38.8%	43.2%	40.8%	32.2%
Rhineland-Palatinate (n = 975)	79.4%	57.4%	48.6%	39.3%	39.3%	41.5%	35.6%	39.9%	38.6%	30.3%
Hesse (n = 885)	73.9%	46.1%	46.4%	45.5%	36.3%	39.6%	37.4%	37.5%	36.3%	31.5%
Saarland (n = 135)	71.1%	49.6%	48.2%	41.5%	38.5%	45.2%	42.2%	40.7%	32.6%	28.2%
Saxony-Anhalt (n = 133)	82%	41.4%	49.6%	39.1%	43.6%	36.1%	30.8%	40.6%	32.3%	39.9%
Berlin (n = 185)	73%	51.4%	53.5%	50.3%	42.7%	37.8%	38.9%	41.1%	42.7%	43.2%
Mecklenburg-Western Pomerania (n = 202)	77.2%	52.9%	35.6%	41.6%	43.6%	39.6%	40.6%	45.1%	30.2%	44.1%
Hamburg (n = 234)	71.4%	47.4%	47.4%	42.3%	38.5%	37.6%	28.6%	31.2%	38.9%	36.3%
Thuringia (n = 146)	76.7%	44.5%	52.7%	48.6%	38.4%	40.4%	48%	34.3%	39%	39%
Schleswig-Holstein (n = 487)	69%	39.8%	42.5%	35.1%	34.1%	37.6%	36.8%	38.4%	33.8%	41.3%
Saxony (n = 122)	84.4%	59.8%	36.1%	49.2%	47.5%	35.2%	37.7%	38.5%	30.3%	29.5%
Bremen (n = 43)	62.8%	39.5%	44.2%	37.2%	44.2%	-	37.2%	25.6%	39.5%	25.6%
Brandenburg (n = 162)	77.2%	50%	38.3%	38.3%	42.6%	47.5%	38.9%	41.4%	38.3%	30.9%

Supplementary table 3: Comprehensive list of the 100 most frequent comorbidities

ICD-10 3-digit	Description	N
C34	Malignant neoplasm of bronchus and lung	13111
I10	Essential (primary) hypertension	9739
E78	Disorders of lipoprotein metabolism and other lipidomes	6744
J44	Other chronic obstructive pulmonary disease	6100
C77	Secondary and unspecified malignant neoplasm of the lymph nodes	5766
C79	Secondary malignant neoplasm at other and unspecified sites	5288
C78	Secondary malignant neoplasm of the respiratory and digestive organs	5225
E87	Other disturbances of the water and electrolyte balance and the acid-base balance	5186
M54	Back pain	5028
J96	Respiratory insufficiency not classified elsewhere	4934
F17	Psychological and behavioural disorders caused by tobacco	4353
E11	Type2 diabetes mellitus	3995
I25	Chronic ischaemic heart disease	3892
R52	Pain, not elsewhere classified	3809
R06	Abnormalities of breathing	3639
J18	Pneumonia, organism unspecified	3637
D38	Neoplasm of uncertain or unknown behaviour of middle ear and respiratory and intrathoracic organs	3529
I50	Heart failure	3352
K29	Gastritis and duodenitis	3149
F32	Depressive episode	3112
H52	Disorders of refraction and accommodation	2966
N39	Other disorders of urinary system	2812
I48	Atrial fibrillation and flutter	2777
K59	Other functional intestinal disorders	2732
N18	Chronic kidney disease	2705
I70	Atherosclerosis	2646
J91	Pleural effusion in conditions classified elsewhere	2638
R63	Symptoms and signs concerning food and fluid intake	2560
M47	pondylo	2495
G47	Sleep disorders	2463
R53	Malaise and fatigue	2407
E79	Disorders of purine and pyrimidine metabolism	2396
N40	Hyperplasia of prostate	2375
R26	Abnormalities of gait and mobility	2364
R11	Nausea and vomiting	2307
K21	Gastro-oesophageal reflux disease	2278
E66	Obesity	2276
J90	Pleural effusion, not elsewhere classified	2273
E86	Volume depletion	2242
E03	Other hypothyroidism	2233
K76	Other diseases of liver	2212
J98	Other respiratory disorders	2024
B96	Other specified bacterial agents as the cause of diseases classified to other chapters	2012

C80	Family history of malignant neoplasm	1983
E04	Other nontoxic goitre	1960
R91	Abnormal findings on diagnostic imaging of lung	1936
D63	Anaemia in chronic diseases classified elsewhere	1868
J43	Emphysema	1867
R32	Unspecified urinary incontinence	1853
I11	Hypertensive heart disease	1834
E14	Unspecified diabetes mellitus	1830
B37	Candidiasis	1807
F45	Somatoform disorders	1754
M51	Dorsalgia	1701
R05	Cough	1689
M17	Gonarthrosis [arthrosis of knee]	1674
D64	Other anaemias	1643
B95	Streptococcus and staphylococcus as the cause of diseases classified to other chapters	1632
I73	Other peripheral vascular diseases	1612
K57	Diverticular disease of intestine	1609
J20	Acute bronchitis	1575
M53	Other dorsopathies, not elsewhere classified	1569
R42	Dizziness and giddiness	1550
R10	Abdominal and pelvic pain	1538
I49	Other cardiac arrhythmias	1528
U50	Motor function restriction	1502
F43	Reaction to severe stress, and adjustment disorders	1490
I83	Varicose veins of lower extremities	1469
R60	Oedema, not elsewhere classified	1452
R04	Haemorrhage from respiratory passages	1439
H26	Other cataract	1408
R64	Cachexia	1406
J45	Asthma	1394
M81	Osteoporosis without pathological fracture	1384
F41	Other anxiety disorders	1376
J40	Bronchitis, not specified as acute or chronic	1373
U69	Other secondary key numbers for special purposes	1339
D70	Agranulocytosis	1327
M16	Coxarthrosis [arthrosis of hip]	1317
R07	Pain in throat and chest	1267
T88	Other complications of surgical and medical care, not elsewhere classified	1261
T81	Complications of procedures, not elsewhere classified	1260
M79	Other soft tissue disorders, not elsewhere classified	1226
H61	Other disorders of external ear	1225
G62	Other polyneuropathies	1218
H35	Other retinal disorders	1216
J95	Postprocedural respiratory disorders, not elsewhere classified	1210
R15	Faecal incontinence	1207
M19	Other arthrosis	1205

N19	Unspecified kidney failure	1179
D62	Acute posthaemorrhagic anaemia	1162
H25	Senile cataract	1157
L89	Decubitus ulcer and pressure area	1150
D68	Other coagulation defects	1145
N17	Acute renal failure	1139
K80	Cholelithiasis	1132
H91	Other hearing loss	1120
M42	Spinal osteochondrosis	1109
D50	Iron deficiency anaemia	1104

Supplementary table 4: Average number of contact quarters with the 10 most frequently consulted outpatient specialists within 4 patient-specific quarters by federal state

	General Practitioner (Mean/SD)	Internist (Mean/SD)	Further internists (Mean/SD)	Other physicians (Mean/SD)	Neurologists (Mean/SD)	No information* (Mean/SD)	Gynaecologists (Mean/SD)	Orthopaedists (Mean/SD)	ENT doctors (Mean/SD)	Surgeons (Mean/SD)
Germany	2.6/1.1	2.3/1.1	2.1/1.0	2.0/1.0	1.8/1.0	1.8/0.9	1.6/0.9	1.5/0.7	1.4/0.7	1.3/0.6
North Rhine-Westphalia	2.6/1.0	2.3/1.0	2.0/1.0	2.1/1.0	1.9/1.0	1.8/1.0	1.6/1.0	1.5/1.0	1.5/1.0	1.3/1.0
Bavaria	2.8/1.0	2.2/1.0	2.0/1.0	2.1/1.0	1.6/1.0	1.8/1.0	1.5/1.0	1.5/1.0	1.4/1.0	1.2/1.0
Lower Saxony	2.6/1.0	2.2/1.0	2.3/1.0	2.1/1.0	1.6/1.0	1.7/1.0	1.6/1.0	1.4/1.0	1.5/1.0	1.3/1.0
Baden-Württemberg	2.7/1.0	2.4/1.0	2.1/1.0	2.1/1.0	1.6/1.0	1.7/1.0	1.6/1.0	1.4/1.0	1.4/1.0	1.4/1.0
Rhineland-Palatinate	2.7/1.0	2.2/1.0	2.0/1.0	1.9/1.0	1.7/1.0	1.7/1.0	1.6/1.0	1.3/1.0	1.4/1.0	1.3/1.0
Hesse	2.7/1.0	2.2/1.0	2.0/1.0	1.8/1.0	1.5/1.0	1.7/1.0	1.5/1.0	1.4/1.0	1.4/1.0	1.3/1.0
Saarland	2.6/1.0	2.0/1.0	2.2/1.0	2.0/1.0	1.8/1.0	1.5/1.0	1.8/1.0	1.4/1.0	1.3/1.0	1.3/1.0
Saxony-Anhalt	2.8/1.0	2.2/1.0	2.2/1.0	1.9/1.0	2.0/1.0	1.7/1.0	1.6/1.0	1.7/1.0	1.4/1.0	1.2/0.0
Berlin	2.5/1.0	2.3/1.0	1.9/1.0	2.0/1.0	1.8/1.0	1.8/1.0	1.4/1.0	1.4/1.0	1.4/1.0	1.5/1.0
Mecklenburg-Western Pomerania	2.8/1.0	2.3/1.0	2.1/1.0	2.2/1.0	2.1/1.0	1.9/1.0	1.4/1.0	1.4/1.0	1.4/1.0	1.6/1.0
Hamburg	2.4/1.0	2.1/1.0	2.1/1.0	2.0/1.0	1.9/1.0	1.8/1.0	1.5/1.0	1.5/1.0	1.4/1.0	1.3/1.0
Thuringia	2.6/1.0	2.3/1.0	2.0/1.0	2.1/1.0	1.9/1.0	1.6/1.0	1.5/1.0	1.2/0.0	1.6/1.0	1.5/1.0
Schleswig-Holstein	2.5/1.0	2.3/1.0	2.0/1.0	1.8/1.0	1.7/1.0	1.8/1.0	1.5/1.0	1.5/1.0	1.3/1.0	1.3/1.0
Saxony	2.7/1.0	2.4/1.0	1.9/1.0	2.2/1.0	2.5/1.0	2.1/1.0	1.4/1.0	1.5/1.0	1.4/1.0	1.2/0.0
Bremen	2.4/1.0	2.5/1.0	2.3/1.0	2.2/1.0	2.5/1.0	1.4/1.0	1.9/1.0	1.6/1.0	1.0/0.0	1.3/1.0
Brandenburg	2.8/1.0	2.0/1.0	2.0/1.0	1.9/1.0	1.4/1.0	1.4/1.0	1.3/0.0	1.5/1.0	1.8/1.0	1.2/1.0

*In the cases with "no information", the data does not contain any information about the physician making the diagnosis

Supplementary table 5: Description of relevant variables

Variable	Description
Lung cancer diagnosis (binary)	• Assured diagnosis of ICD-10-C34 in the outpatient sector or main or secondary diagnosis of ICD-10-C34 in case of discharge in the inpatient area<br>• Documentation of the diagnosis in the outpatient sector by 2 different physicians in the index quarter or mention of the diagnosis by the same physician in two different quarters<br>• One-time documentation of the diagnosis in the inpatient area<br>• Inclusion of incident lung cancer patients from 1st quarter 2013, preliminary observation period (validation period) is 4 quarters, follow-up period 2 quarters
Age (continuous)	• Age will be calculated as the age on 31st of December in the recent year and as the age on 31st December 2017 for the five-year prevalence. Patients will be assigned to three age groups (younger than 65, 65-85, older than 85)
Gender (binary)	• Gender of patients (male/female) at the moment of cohort entry
Comorbidities (binary)	• Assured outpatient diagnosis (3-digit) and/or inpatient primary and/or secondary diagnosis (3-digit) according to ICD-10-GM<br>• Exclusion form O and Z classification (O = Pregnancy, Birth, puerperium, Z = facts influencing the state of health and lead to the use of healthcare)<br>• Comorbidities are identified during four quarters:<br>• quarter prior to lung cancer diagnosis, quarter in which lung cancer diagnosis was made and two quarters after lung cancer diagnosis
Specialist groups (binary)	• Lifelong physician identifier number (LANR 8th & 9th digits give information about specialist group)<br>• Link "outpatient diagnoses" - contains information Lifelong physician identifier number (who has made outpatient diagnosis), i.e. Lifelong physician identifier number for first outpatient diagnosis<br>• Patients who have been diagnosed by two different outpatient physicians in the index quarter are counted twice (this means that it is not clear who was diagnosed first)
Number of quarters to specialist groups (continuous)	• Capture of the lifelong physician identifier number (LANR) for each individual billed service figure (EBM figure)<br>• Alternative: billing code according to EBM - clear allocation of a case to the exact specialist group of a physician, of whom most billing rates are available in the context of a treatment case.
Patient's place of living	• On the level of federal states
